# Successes and lessons learned from a mobile health behavior intervention to reduce pain and improve health in older adults with obesity and chronic pain: a qualitative study

**DOI:** 10.3389/fpain.2024.1340400

**Published:** 2024-04-25

**Authors:** Amber K. Brooks, Abha Athawale, Virginia Rush, Abigail Yearout, Sherri Ford, W. Jack Rejeski, Ashley Strahley, Jason Fanning

**Affiliations:** ^1^ Department of Anesthesiology, Wake Forest University School of Medicine, Winston-Salem, NC, United States; ^2^ Department of Health and Exercise Science, Wake Forest University, Winston-Salem, NC, United States; ^3^ Department of Social Sciences and Health Policy, Wake Forest University School of Medicine, Winston-Salem, NC, United States

**Keywords:** mHealth (mobile health) technology, weight loss, mindfulness, social support, pain management, physical activity, chronic pain

## Abstract

**Introduction:**

Chronic pain is a prevalent issue among older adults in the United States that impairs quality of life. Physical activity has emerged as a cost-effective and non-pharmacological treatment for chronic pain, offering benefits such as improved physical functioning, weight loss, and enhanced mood. However, promoting physical activity in older individuals with chronic pain is challenging, given the cyclical relationship between pain and sedentary behavior. The Mobile Intervention to Reduce Pain and Improve Health (MORPH) trial was designed as an innovative, mobile health (mHealth) supported intervention to address this issue by targeting daylong movement, weight loss, and mindfulness to manage pain in older adults with chronic multisite pain. The objective of this paper is to provide the result of a qualitative analysis conducted on post-intervention interviews with MORPH participants.

**Methods:**

At the conclusion of the MORPH study, 14 participants were interviewed regarding their experience with the program. All interviews were conducted by phone before being transcribed and verified. A codebook of significant takeaways was created based on these accounts. Summaries were further synthesized into themes using the principles of thematic analysis.

**Results:**

Three key themes of the MORPH intervention emerged from the qualitative interviews: MORPH technology (smart scales, Fitbit, MORPH Companion App) facilitated program adherence and accountability; MORPH intervention components (food tracking and mindfulness activities) facilitated program adherence and awareness, respectively; and, group meetings provided motivational support and accountability. Mobile health technologies, including a dedicated MORPH app, facilitated self-monitoring strategies, helped to break the cycle of old habits, and provided participants with immediate feedback on successes; however, technical issues required timely support to maintain engagement. Food tracking contributed to adherence and accountability for weight loss. Mindfulness activities increased participants’ awareness of anxiety provoking thoughts and pain triggers. Finally, social support via group meetings and connection, played a crucial role in behavior change, but participants noted consistency in the delivery medium was essential to fostering genuine connections.

**Conclusion:**

Overall, the study results highlight the key considerations related to program technology, intervention components, and the value of social support that can help to guide the development of future interventions similar to MORPH.

## 1 Introduction

Chronic pain affects 30.8% of older adults in the United States (U.S.) (1) and is a leading cause of anxiety, depression, disability, and sleep disturbances with an estimated annual treatment cost that is almost 30% higher than cancer and diabetes combined (2). The importance of identifying effective interventions for pain management has increased as the median age in the U.S. continues to rise. This paper describes qualitative analyses of interview data collected in conjunction with a novel treatment for chronic pain in older adults with obesity, the Mobile Intervention to Reduce Pain and Improve Health (MORPH) trial.

Clinicians have historically recommended that rest is the best source of treatment and recovery for pain (3) with pharmacologic management being a common first line therapy, despite concerns regarding their deleterious effects on health. Older adults are more susceptible to the side effects of pain medication due to the higher prevalence of coexisting chronic conditions such as renal disease and the rising prevalence of opioid misuse disorder. Older adults have recently been described as the unseen face of the opioid epidemic with opioid misuse identified during emergency department visits for this population experiencing a 220% increase between 2006 and 2014 (4). Notably, physical activity and exercise programs are increasingly being offered as an accessible treatment option for pain management which also improves physical functioning (5).

Successful pain self-management necessitates that clinicians provide their patients with appropriate instruction, education, and support; it is also recommended that movement be incorporated into this pain management plan. Goh and colleagues (6) conducted a systematic review and identified 77 randomized control trials targeting pain management of knee and hip osteoarthritis via exercise-based interventions and found significant improvement in pain, function, performance, and quality of life. These results were heterogeneous and suggested that exercise was most effective among participants less than 60 years old. Generally, interventions focused on enhancing physical activity do so via traditional structured exercise programming (i.e., physical training done for the explicit purpose of enhancing health or fitness, often in discrete lengthy bouts and often through limited types of activity such as walking, jogging, or using exercise equipment). It is notable that such traditional programming may not be the optimal strategy for managing pain in the long term. Many find exercise aversive and are unlikely to sustain it. Furthermore, exercise programming promotes the view of physical activity as a discrete behavior occurring once per day, raising the risk of overexertion and hyperalgesia while contributing to compensatory sitting (7). Recognizing these limitations, researchers across disciplines have begun to shift toward activity programs that focus on achieving health-enhancing levels of activity by engaging in diverse and enjoyable activities across the day, thereby breaking up sustained sitting.

Adopting a novel approach to health behavior change, such as promoting movement across the day, requires the development and refinement of new strategies to target constructs associated with successful behavior change, such as behavioral self-regulation and self-efficacy. Whereas exercise programming requires individuals to muster substantial motivation several times per week, a program focused on moving throughout the day requires a lower level of motivation that is sustained continuously in the face of diverse daily barriers. Thus, providing social support, fostering objective self-monitoring, and cueing the individual to their successes (key inputs to self-efficacy) must be done continuously. To this end, mobile health (i.e., mHealth) technologies, such as computers, smartphones, tablet computers, and wearable devices, have come to play a crucial role in activity promotion programs in recent years. They offer solutions to several pressing challenges in the delivery of behavioral interventions. They can provide objective self-monitoring data in near-real time to guide goal setting, which is crucial for effectively self-regulating behavior (8). Rather than relying on infrequent and brief intervention meetings to facilitate social support and accountability, mHealth technologies can offer opportunities for synchronous or asynchronous social connection at any time. This is crucial as social connection lies at the root of human health and successful behavior change (9). Finally, mHealth technologies can alert participants of their behavioral successes in real-time, assist in creating awareness of dysfunctional habits at the root of inactivity, cueing the need to employ strategies that are designed to break the cycle of engrained habits, as well as enhance the timeliness and specificity that are required elements of self-efficacy-enhancing feedback (10).

Importantly, realizing the promises of mHealth tools in the context of physical activity research in aging requires careful development driven by the science of behavior change and principles of user-centered design. The health effects of an in-person exercise intervention hinge upon participants’ attendance at intervention sessions. Similarly, the extent to which mHealth tools can support behavior change is tied to whether individuals utilize the toolset, which is driven by the individual’s perceptions of the usability and usefulness of the tools. The application's perceived usefulness encompasses whether the behavior it supports is valued by the individual and whether the digital toolset provides support that is likewise tangible and valuable to the individual. For instance, does the toolset provide individuals with an enjoyable sense of connection with others in the group, is the visual feedback informative and intuitive, and is the cueing of successes exciting and motivating? Do they find the social tools cumbersome and awkward, the feedback unintuitive or more accessible in another form (e.g., on the screen of a wearable), or the cues disconnected from their associated behavioral successes?

Previous work has demonstrated that physical activity is a cost-effective non-pharmacological treatment method for individuals with chronic pain, showing beneficial results on physical functioning, sleep, and mood, especially when activity programming is individually tailored (11). Unfortunately, promoting physical activity in any population is a challenge, and this is especially true for those with chronic pain. Indeed, chronic pain and inactivity operate cyclically, as low levels of physical activity can be a consequence of and/or exacerbate pain (12). To this end, we conducted the MORPH trial: an iterative pilot randomized controlled trial targeting physical activity, weight loss, and mindfulness to manage pain in older adults with chronic multisite pain and obesity (13, 14). MORPH was unique as it was a mobile health (mHealth)-supported intervention that explicitly attempted to act upon the cyclical relationships between pain, eating, and activity behaviors.

Bandura (10) offers a useful lens for understanding the value of user-centered design for creating valued digital health tools that produce sustained use. Bandura notes that behaviors arise from interactions between the traits of an individual, the traits of targeted behaviors, and traits of the social and built environment. From this perspective, the design of a novel digital health toolset must be iterative and must involve members of the target population, as their aesthetic preferences, intuitions, and desires necessarily differ from those of other populations. Similarly, the extent to which that individual values the behavior that the toolset is meant to promote and the extent to which the toolset tangibly supports the behavior will affect whether a toolset is used. Notably, it may be expected that during periods of skill acquisition (e.g., the development of an intuitive understanding of one's activity patterns), the use of a toolset may be higher than in periods of behavioral maintenance. Finally, aspects of the individual's built and social environments will affect the individual's expectations of the form and function of a digital health app, including how it supports behavior change. Whether an individual receives sufficient training to use a digital health tool, has previously developed technological proficiency through academics or the workforce, or is surrounded by individuals who engage in a targeted health behavior, will inform the design languages that individuals find intuitive, the extent of their reliance on technological support (and the ways this support should be provided to instill efficacy toward technology usage), and the individual's daily technology usage habits.

The MORPH iterative pilot randomized controlled trial centered on the development of a hybrid in-person and remote physical activity behavioral intervention for older adults with chronic multisite pain. The MORPH activity intervention was focused on the accrual of daily steps by moving often throughout the day and avoiding sustained periods of sitting. This approach to activity promotion is novel; notably, there are few mHealth-supported physical activity interventions for older adults with chronic pain (15). Therefore, MORPH emphasized user-centered design consisting of two phases. The first included a series of N-of-1 refinement trials and the second a 12-week randomized controlled trial wherein participants were interviewed on completion (13). What follows is a description of the MORPH study and a qualitative exploration of participant interviews focused on understanding how the structure and function of the MORPH program and associated app affected participants’ perceived value of a daylong movement intervention. We believe that sharing these findings will assist others in designing useful and usable mHealth tools for older adults with chronic pain.

## 2 Methods

### 2.1 Participants

The methods and CONSORT diagram for MORPH were previously published (13, 14). Participants were recruited between March 2018 and October 2019. Individuals eligible for the study were between 55 and 85 years of age with a body mass index (BMI) of 30–45 kg/m^2^, considered low active, were weight stable for the previous 6 months, had no contraindication to exercise, owned a smartphone, and had pain in at least two of the following places in the previous 3 months: back, neck, shoulders, hips, or knees. Based upon these criteria, in total 15 individuals were randomized to the MORPH condition and 14 of the 15 agreed to be interviewed. The participants had an average age of 69.70 ± 4.12. Most of these participants were female (71.4%) and white (92.9%). The majority of participants completed up through a college level degree (71.4%), with some having completed a postgraduate degree (21.4%), and 7.1% having completed up through high school. Finally, participants BMI and Short Physical Performance Battery scores were 35.79 ± 4.19 and 10.00 ± 1.47, respectively.

### 2.2 Intervention procedures

The MORPH pilot trial was a two-phase pilot study primarily focused on the iterative development of a smartphone app-supported group-mediated behavioral intervention that was designed to promote physical activity throughout the day and caloric restriction in older adults with chronic pain. During Phase 1, a series of N-of-1 design studies were conducted with participants to identify and address technical and usability issues of the smartphone app, and qualitative interviews were performed to address these shortcomings. Phase 2 comprised a 12-week randomized controlled pilot study of the holistic intervention versus a wait-list enhanced care control. This was succeeded by follow-up interviews evaluating participant preferences, use, and usability challenges. Intervention groups concluded in October and December 2019, and January 2020. Delayed intervention groups concluded in March and June 2020. Only those who were randomized to the intervention in Phase 2 were approached for an interview.

### 2.3 Active intervention group intervention procedures

#### 2.3.1 Weekly group meetings

Participants attended weekly meetings in small groups led by behavioral professionals including a nutritionist and behavioral interventionist. These sessions were informed by social cognitive and self-determination theories, mindfulness-based relapse prevention, and principles of group dynamics (10, 16, 17). Group sessions, a crucial tool for behavior change, offer accountability, a forum for collaboratively addressing barriers, and generating movement repertoires, and modeling successes. Each session included a group bonding activity, educational content related to pain, the development of skills required to successfully change behavior, a lesson on the role of weight management and activity for managing pain, and a practical mindfulness skill. These sessions also offered an opportunity for participants to collaborate with group leaders to set new caloric restrictions and step goals. Regarding caloric restriction goals, participants aimed to achieve approximately 3% reduction in body mass over the 12-week program via caloric restriction. This was monitored via food logging; participants were taught how to track intake and could use any tool they found convenient. Participants aimed to increase average daily steps across the 12-week program by approximately 20% weekly, tailored to the individual and their progress. Emphasis was placed on distributing these steps across the day. Maintenance goals were set when participants achieved 10,000 steps (18). The first three sessions were held in person with the intention of fostering group bonding, and the final nine weekly sessions were conducted via video conference on WebEx (Cisco) software. The study transitioned to an entirely online format, following the start of the COVID-19 Pandemic in March 2020.

#### 2.3.2 Study devices

Participants were provided with a Fitbit device to collect data on minute-level stepping behaviors and to transmit these data to the study-specific app in near real-time. These devices were chosen as they provide programmatic access to minute-level stepping data (which are key to the MORPH app feedback as described below) and offer high compliance amongst participants. Participants also received a BodyTrace cellular weight scale and were encouraged to weigh daily throughout the study period, with the resulting data being accessible immediately within the study mHealth application.

#### 2.3.3 The MORPH Companion App

MORPH participants received the *MORPH Companion App–*a set of digital health tools designed to act as a companion to the MORPH group-based behavioral intervention. The *Companion App* served three key intervention functions: (1) to provide access to social support from peers and group leaders between weekly group meetings via asynchronous chat; (2) to help participants develop an intuitive understanding of their habitual patterns of movement throughout the day; and (3) to cue successes in near real-time to support self-efficacy. As noted above, Fitbit data were integrated in near real-time to provide visual and numeric feedback to the participant and researcher. The key feedback mechanism was a daily “timeline bar.” Here, movement patterns were visualized on a color-coded bar such that periods of activity were displayed in green and inactivity displayed in blue. Participants were instructed to view the bar frequently throughout the day and aimed to achieve a “tree rings profile,” which is a visual metaphor to describe a day with frequent stripes of green movement and few sustained periods of blue inactivity; thus, making it resemble tree rings. Additionally, to disincentivize achieving one's daily step goals in a single bout and then engaging in sustained sitting, participants received a daily “periodic” step goal. For this, the participant's daily goal was subdivided into three daily periods (morning, midday, and night) wherein they could achieve up to 45% of their overall daily goal. Steps exceeding that 45% within a period did not count toward the overall daily periodic step goal; therefore, requiring some amount of participant movement during each of the three periods. Finally, to support self-efficacy for achieving daily activity goals via mastery experience, participants received “mastery badges” (19). These are highly specific graphic badges that are explicitly tied to programmatic goals (e.g., maximizing a period, achieving daily periodic step goals, achieving weekly activity goals) and are released in near real-time to encourage participants to recognize and savor successes. In addition to the Companion app, participants were offered varied opportunities for self-monitoring food intake, including paper logs and smartphone applications such as MyFitnessPal or Fitbit.

### 2.4 Wait-list control group intervention procedures

Those randomized to receive the wait-list control received an enhanced usual care control program. These individuals received the Fitbit activity monitor and BodyTrace scale to account for any short-lived behavioral impact of device provision. Upon completion of the 12-week study period, they were offered the opportunity to engage in the MORPH program.

### 2.5 Qualitative interview procedures

Study participants were contacted between June and August 2020 for a post-intervention interview. All 15 participants of the intervention condition were approached for an interview and 14 consented (93% of MORPH intervention participants in Phase 2). Interviews, data collection, and data analyses were performed by the Wake Forest University School of Medicine Qualitative and Patient-Reported Outcomes (Q-PRO) shared resource group staff. The Q-PRO staff provide methodological expertise in qualitative research and patient-reported outcomes for clinical studies. The study coordinator asked participants who consented if they preferred to be interviewed by telephone or by videoconference; all participants opted for interviews by telephone. Participants were then contacted by Q-PRO staff, scheduled, and interviewed. Using an institutional review board-approved interview guide (see Supplementary Material S1), the Q-PRO staff collected feedback on recruitment, study staff performance, the study app, programmatic components, and participants’ overall study experiences. Specifically, participants were asked about what they found helpful and not helpful within these domains to guide the development of future MORPH iterations. Interviews ranged from 21 to 60 min, and the average length was 34 min. All interviews were audio recorded and transcribed verbatim. After verifying the accuracy of all transcripts, two Q-PRO staff developed a codebook to highlight information specific to programmatic components in addition to other components mentioned by participants, such as accountability and motivation. We assessed saturation by determining the point within the dataset at which no new relevant ideas emerged (20). We organized the data in chronological order and documented the progression of concept identification within each interview. We employed a stopping criterion of three transcripts, meaning that once no new concepts were identified in the data, we reviewed another three transcripts for confirmation (21). Using this method, saturation was reached at 12 interviews. All data were managed with the ATLAS.ti software and coded using a consensus-based coding approach, which prioritized coding consistency and agreement. Triangulation, a qualitative research strategy to ensure the reliability and validity of qualitative results, was employed in this study. Participant feedback collected through surveys and self-reported issues with study equipment (e.g., device issues) were used to triangulate the interview data. The two Q-PRO staff independently coded the transcripts in groups of 2–4 transcripts and met after each group of transcripts was coded to compare. As transcripts were reviewed, the codebook was adjusted to best capture the meanings and significant takeaways from participants. Discrepancies in coding were also discussed and resolved throughout the process of analyzing transcripts. Summaries were further synthesized into themes using the principles of thematic analysis.

## 3 Results

### 3.1 Participants

Participant characteristics are displayed in Table 1. 92.9% of participants were white and 71.4% were female. The average age of the participants was 69.7 ± 4.12 years of age. As a part of the study requirements, all participants reported having chronic pain in either the back, neck, hip, knee, or shoulder.

**Table 1:** MORPH Topics, Themes, and Illustrative Quotes.

Topics	Themes	Illustrative Quotes
MORPH technology	MORPH Technology (smart scales, Fitbit, MORPH Companion App) facilitated program adherence and accountability • *Fitbit and MORPH App rewards systems provided motivational support* • Technological challenges with the scales, Fitbit, and MORPH App	*“The fact that I had the Fitbit reminded me to get out of the chair.” “…when it would graph my movements and stuff, that definitely helped me understand where I was hittin' it, ‘cause [there] would be periods of when I wasn't really movin’, and it motivated me to get up and make sure I did it more.” “The part that was difficult is we were supposed to be able to share some things. I forget what it's called on that particular part of the app, that you could remain in contact with the group, you could share recipes or whatever. That part was not working. I couldn't get it to scroll down so I could read part of it. It was really hard to add things to it.”*
MORPH intervention components	Food tracking contributed to adherence and accountability Mindfulness activities improved awareness of anxiety provoking thoughts	Food tracking *“opened my eyes to what needed to change.” “I learned because I've never counted calories. I never counted fat grams. I never knew how to. I just didn't bother to learn ‘cause I've never had to. So, the nutritional part with Bev was fantastic for me. It was very time-consuming to look up in the little book every single thing you put in your mouth all day long. That was a complaint that we all had, but we all understood, or at least I did. I understood the reasoning behind it and the education that I derived from having someone hold my feet to the fire and say, ‘Okay, you have to do this so you can learn.’ That was good.” “…when you start beating yourself up, ‘All right, you ate too much yesterday, and you're way over your allotment,’ or ‘You didn't walk enough yesterday,’ or ‘Darn you. You drank water, but you forgot to record it,’ all that kinda stuff. If you just stop that tape in your head-and I think that's what the mindfulness thing did.”*
Group meetings	Group meetings provided motivational support and accountability Challenges with the virtual format inhibited some participants from fully accessing social support from group leaders and other participants	*“I liked it if anybody had recipes that they wanted to share, because you come up with different ideas ‘cause you don't wanna keep eatin’ the same stuff all the time. Or ideas on what they did for exercise or what they did to make sure they got up and did it.” “There were times that, when I was going to get onto the Webex meeting, that I could hear them, but I could not see them. I knew that it was not my computer or my setup, because I work from home and have Webex meetings for my company with no issues. Somewhere, there's a glitch with how it's being done.”*

Three key themes of the MORPH intervention emerged from the qualitative interviews: MORPH technology (smart scales, Fitbit, MORPH Companion App) facilitated program adherence and accountability; MORPH intervention components (food tracking and mindfulness activities) facilitated program adherence and awareness, respectively; and, group meetings provided motivational support and accountability. Illustrative quotes provide context and support for the themes.

### 3.2 Major themes

Three major themes of the MORPH intervention (Table 1) emerged from the qualitative interviews: MORPH technology (smart scales, Fitbit, MORPH Companion App) facilitated program adherence and accountability; MORPH intervention components (food tracking and mindfulness activities) facilitated adherence and awareness, respectively; and, group meetings provided motivational support and accountability.

### 3.3 MORPH technology

#### 3.3.1 Smart scales

Participants were provided with scales and asked to track their weight throughout the course of the intervention. The smart scales were found helpful by most participants for regularly tracking their weight as it kept them accountable for their weight loss. For example, one participant indicated that “it's useful to see where you're at in your journey,” and another individual thought that the scale was “a catalyst for you to stay the course.”

The most noted obstacle, voiced by about half of the participants, was getting the scales to log and transmit the correct weight to the study team. One individual in particular noted frustration with the weight logging aspects of the scales saying, “they would read my weight… [but] it never would record my weights.” It is notable that at the time of the study, BodyTrace smart scales utilized the AT&T cellular network, and homes with poor connectivity often resulted in loss of data during transmission. Another participant had a “problem balancing on those scales,” and felt unsafe, stating there was “nothin” to hang on to or catch if [they fell]” while taking their weight.

#### 3.3.2 Utility and integration of the Fitbit and MORPH Companion App

The Fitbit device and app were used by participants to track steps each day and participants generally reported they were beneficial. One participant explained that “it would graph my movements and stuff, that definitely helped me understand where I was hittin’ it cause [there] would be periods of when I wasn't really movin, and it motivated me to get up and make sure I did it more.” Additionally, one participant found that the graphical display of their movement throughout the day was more helpful than the Fitbit app alone and allowed them to learn what times during the day they needed to move. The delivery of the movement data on the MORPH app was appreciated more overall, as the feedback provided insight into long periods of inactivity.

The MORPH Companion app that was developed for the intervention had specific components that several participants cited as being helpful. Specifically, the reward system implemented on the app, motivated a few of the participants to increase their movement throughout the day. One stated, “it reminded you that there were things to do, that you could [do] something about your weight…[and] about your pain.” Another participant reported “it was nice to see that you got this many chair rewards by being up and down too.”

The most commonly cited challenge with the Fitbit, reported by a third of the participants, were issues with the device not working correctly or the band breaking. Additionally, some expressed difficulty managing both the Fitbit and Companion App. One participant explained, “the MORPH [app] doesn’t work unless you got the Fitbit running,” and another found that the Fitbit removed the need for the MORPH app, stating “I really had what I needed with the Fitbit and the MyFitnessPal,” which the participant used to self-monitor dietary behaviors.

### 3.4 MORPH intervention components

#### 3.4.1 Food tracking

Participants noted that the food tracking, regardless of the tool they used, elucidated areas of their diet that they could improve upon. One participant commented, “figuring out your daily gram intake, your average fats and proteins…that's what really opened my eyes to what I needed to change about my diet.” Participants expectedly viewed food tracking to be “a little bit more time-consuming and difficult…[it] wasn't bad, though, because that was where you really figured it out.”

#### 3.4.2 Mindfulness activities

Mindfulness activities helped multiple participants reduce anxiety and negative self-talk when they fell behind in their diet and exercise goals. One participant noted that “you just need that relaxation. It was like you don't give yourself permission, but the mindfulness thing helped you get there.” Furthermore, another participant felt as though mindfulness made them “more aware of days that were better, and that exercise was key. Keeping up with daily exercise was key to keepin’ the pain levels down.” As mindfulness practices were new to many participants, some found it difficult “to go off and sit in a corner and meditate for an hour, or even five minutes.”

### 3.5 Group meetings

The group meetings led by the behavioral interventionist and nutritionist were motivational and helped participants remain accountable. One participant thought these meetings were “quite good because it was reinforcing [and] always positive feedback. It made you feel good that you were doing something that was… good in their eyes.”

A common challenge expressed by participants was the transition in modalities from in-person to virtual meetings, which some felt interrupted the development of interpersonal connection following the three weeks of in-person meetings. One participant explained that once the intervention transitioned, “we didn't have a lot of players in the second portion of the program,” indicating the shift in participant involvement. Additionally, participants expressed difficulties with the video conferencing software, WebEx, that was used and noticed “somebody would get up and go to another room. Somebody's kid was in the other room. I just found all that distracting.”

## 4 Discussion

Herein, we aimed to identify the key participant takeaways from the MORPH trial and to examine possible ways to evolve the protocol for future iterations. The main goal in the pilot phase of MORPH was to examine the feasibility of delivering a home-based technology-driven intervention to older adults with chronic pain. Lessons learned from the emerging themes and design considerations for future intervention development are discussed in-depth below.

### 4.1 MORPH technology

Mobile health technologies are useful for providing feedback that can help to raise awareness of maladaptive habits while highlighting successes as they occur, thus enhancing self-efficacy via mastery. Feedback suggested that the technologies utilized for intervention delivery in MORPH, especially the Companion app, provided beneficial feedback and aided in self-monitoring goal progress. Some participants noted challenges with connectivity—especially for cellular-enabled scales—and complexity associated with operating several smartphone applications concurrently. Additionally, audio issues were noticed on WebEx, such as muting speakers and excessive background noise, which hindered comfortable conversation between participants (22). These findings align with the systematic review completed by Changizi and Kaveh (23), which demonstrates the utility of mHealth as a useful platform in facilitating behavior change. MORPH participants also identified the need for support when technological issues arose and the inclusion of goal setting for incremental success as well as personalized, timely feedback (24, 25).

#### 4.1.1 Design considerations

Key to uptake and use of mHealth technologies in any population is a supportive infrastructure that facilitates ongoing technology support to address issues with connectivity and complexity. This personnel requirement is an important bottleneck in the broad scale implementation of behavioral mHealth tools. To address this challenge in MORPH-II, we piloted a pre-medical student lay coaching model whereby pre-medical undergraduate students received behavioral and technology training and then served as supportive staff to the intervention. This meets the unique needs of the rapidly-growing body of 55,188 prospective pre-medical students who desire patient and participant interactions, and offers a low-cost model to deliver coaching and to support participants' needs (26). Furthermore, the transition to Zoom as the primary video conferencing platform addressed usability and audio difficulties present in WebEx. Finally, we implemented a second technology orientation for MORPH-II, ensuring that remaining questions were addressed prior to the start of the intervention. From a design consideration perspective, working with individuals of any specific demographic requires personalization of mHealth technologies to the individual, as well as support to deal with expected technical difficulties.

### 4.2 MORPH intervention components

Mindfulness increases awareness to prevent relapse in participants when study mechanisms are understood. Feedback suggests that engaging in the mindfulness exercises utilized in MORPH aided participants in connecting their daylong movement levels and decreased chronic pain to instill lasting behavior change. Perhaps, unsurprisingly, not all participants resonated with the mindfulness activities, as exemplified by one participant noting difficulty in finding time and space to engage in mindful meditation. Future studies should include participant-led discussions of barriers and solutions to engage in mindfulness activities. The randomized control trial conducted by Bowen et al. (17) supports that engagement in mindfulness increases awareness of reactions, motivations, and triggers to counter reactive behaviors and instill skillful responses, highlighting the utility of mindfulness practices for preventing relapse to prior unhealthy behavioral patterns (27).

#### 4.2.1 Design considerations

As mindfulness is a complex practice, it requires explicit and transparent delivery to promote behavior change. Based upon lessons learned in MORPH, MORPH-II leveraged frequent brief individual coaching contacts to encourage the use of mindfulness techniques; an approach that offers greater opportunity to tailor mindfulness practice to the individual's preferences and barriers.

### 4.3 Group meetings

Social support is valuable for encouraging movement behavior change, but consistent delivery is necessary. Emotional support, often from a spouse, family member, or peer, can motivate certain health-enhancing behaviors (28). Within the MORPH trial, participants found that group meetings were motivating due to the positive feedback. The transition in modalities from in-person to virtual delivery, however, hindered personal connection between participants. Lewis et al. (29) found in a study with clinical patients during the COVID-19 pandemic that many participants were reluctant to make the transition from face-to-face treatment to virtual meetings. Previous evidence supports video conference as an effective way to meet, but as with any technology, the use of video conference software should be approached systematically and include opportunities to develop comfort and social bonds to prevent “Zoom Fatigue” and other medium-specific barriers (30).

#### 4.3.1 Design considerations

Results suggest that consistency in the meeting medium is important for the development of social support, especially in partially or fully remote intervention protocols. This was counter to expectations for the MORPH trial results, as we expected meeting in person initially would establish strong relationships before meeting virtually for the remainder of the intervention. Thus, MORPH-II we implemented a fully remote delivery to explore the feasibility and acceptability of this approach (22).

### 4.4 Limitations and additional considerations

It is important to interpret the findings of this study in the context in which they were collected. Most participants were white and female and, therefore results may not be generalized outside of this demographic. Second, we implemented brief interviews that aimed to capture limitations to the design and implementation of the protocol and mHealth technologies to facilitate additional refinement as needed. Therefore, we received limited information on aspects of the protocol and digital toolset that participants found most beneficial for modifying their behaviors. Future implementations would benefit from the recruitment of a diverse sample and a more inclusive qualitative research component. Lastly, the interviews were conducted more than a year after the intervention was completed, which may have introduced recall bias.

## 5 Conclusions

Chronic pain is an increasingly prevalent problem across older populations that promotes sedentary behaviors and, thereby, impedes quality of life. MORPH aimed to manage pain in a lasting and accessible manner through a daily movement intervention that employed group mediated sessions and a mHealth technology toolset. Participant feedback regarding these interventions, collected via qualitative interviews, serves to bolster the development of future iterations of MORPH. In addition, lessons learned through this qualitative analysis have the potential to impact the delivery of prospective long-term behavioral and remote interventions. Specifically, findings emphasized the importance of consistency in meeting modality (i.e., either in-person or via video conference), the critical role of technical support tailored to the target population, and the value of mindfulness-enhancing strategies in the context of challenging behavior change.

## Data Availability

The raw data supporting the conclusions of this article will be made available by the authors, without undue reservation upon request.
