# Supplementary material for: Successes and lessons learned from a mobile health behavior intervention to reduce pain and improve health in older adults with obesity and chronic pain: a qualitative study
Source: Front Pain Res (Lausanne). 2024 Apr 25;5:1340400. doi: 10.3389/fpain.2024.1340400 (PMC11079154; doi:10.3389/fpain.2024.1340400)
Supplement: Supplementary file 1 [file Datasheet1.docx]

**Interview Guide**

Note to interviewer: This guide is structured into four sections (introduction, recruitment, componentry, and wrap-up). Within each section are key discussion points (enumerated) followed by additional prompts (bullets-optional) that can be used to foster conversation.

**PID: _______________ Date: _______________ Start Time: ________________**

**Introduction**

- Thanks for your time
- Introductions
- We are very grateful for your participation in the pilot phase of our study. The goal of the study was to try out tools that we hoped would help people with pain move more throughout the day and lose weight. The purpose of today’s conversation is to help our study team learn from your experience in the program. We would like to know more about what you found to be helpful and not helpful, and what you recommend that we do to improve the study.
- I consider you as an expert consultant on your experience in the study and how we can best plan our next step, and so anything that you share will be highly valuable to us.
- This interview will last no longer than an hour, and I will audio record our conversation so we can go back and document your feedback. If at any point you would like to take a break, skip a question, ask a question, or stop the interview, please just let me know.
- Do you have any questions for me before we start?

**MORPH Recruitment**

First, let’s talk about the very beginning of your involvement.

1. How did you hear about the study?
2. Why were you interested in participating?
3. In what ways did this program meet, exceed, or fail your expectations? Please explain.
   - What were your expectations?
4. Tell me about your experience with members of the study team who discussed procedures and worked with you to complete questionnaires and other tests.
   - Were they responsive? How?
   - Did they seem knowledgeable?

**MORPH Components**

Next, let’s talk about your time with the MORPH program itself. We had several things we were interested in trying out in this program, so I’d like to chat with you about each of them.

1. First, please tell me about your experience completing the daily surveys on your phone.
   - How easy or difficult did you find them to complete? Please elaborate.
   - What effects did completing the surveys have on your thoughts or perceptions about your pain?
2. Tell me about your experience with using the scale at home.
   - Why was/wasn’t it useful to track your weight over time?
3. We asked you to use the MORPH app throughout the program (this is different from the Fitbit App). How often did you try to use the app? Why?
4. Tell me about what you liked, or found useful about the app.
   - Do you feel as though the app helped you to better understand your physical activity levels? Why or why not?
5. What about the app did you dislike, did not understand, or not find useful?
6. What was the app missing? In other words, what did we fail to include that you would have found helpful?
   - Were there things that you wished were in the app that we did not have in the app?
7. We included a series of podcasts in the app. How many do you think you listened to?
   - If zero or none, “Why not?”
   - What did you think of the podcasts?
8. We included a series of cartoon videos in the app. How many videos would you guess you watched?
   - If zero or none, “Why not?”
   - What are some things you liked and disliked about the cartoon videos?
9. In addition to using the app, we asked you to meet in a group each week. First, we had you meet in person, and later on WebEx. What was your experience in the group meetings?
   - What did you like and dislike about the group meetings?
   - If you preferred one style over the other, in-person or WebEx, tell me why.
   - Do you feel like you meaningfully connected to others in your group?
     - Tell me more about that.
     - Which format was more helpful in establishing that connection – in-person or WebEx?
     - Did you feel connected to the leader? Why/Why not?
10. Tell me more about your group leader.
    - How clearly do you feel they explained each aspect of the program?
    - What are some things the leader could have done to help you feel supported in becoming more active?
    - If you had been the group leader, what would you have done differently?

**MORPH Program Overall**

**Let’s conclude by talking about the program overall**

1. Overall, what did you think about the MORPH program?

Since your involvement in MORPH –

1. Do you think differently about pain and how it relates to physical activity? Please explain.
2. Do you think MORPH has resulted in any long-term changes in your life? Please explain.
3. If you were to describe your experience with MORPH to someone who has never heard of it, how would you describe it?
4. Other than what we’ve already discussed, what additional suggestions for improving the MORPH program do you have for us? These changes could be related to the app, personnel or any of the study components.
5. And finally, is there anything else you’d like to tell us that we didn’t ask about?

Thank you for sharing your thoughts with me today!

**End Time: ________________**
