# Supplementary material for: Successes and lessons learned from a mobile health behavior intervention to reduce pain and improve health in older adults with obesity and chronic pain: a qualitative study
Source: Front Pain Res (Lausanne). 2024 Apr 25;5:1340400. doi: 10.3389/fpain.2024.1340400 (PMC11079154; doi:10.3389/fpain.2024.1340400)
Supplement: Supplementary file 2 [file Table1.docx]

Table 1: MORPH Topics, Themes, and Illustrative Quotes

| **Topics** | **Themes** | **Illustrative Quotes** |
| --- | --- | --- |
| MORPH Technology | MORPH Technology (smart scales, Fitbit, MORPH Companion App) facilitated program adherence and accountability   - Fitbit and MORPH App rewards systems provided motivational support - Technological challenges with the scales, Fitbit, and MORPH App | *“The fact that I had the Fitbit reminded me to get out of the chair.”*  *“…when it would graph my movements and stuff, that definitely helped me understand where I was hittin’ it, ‘cause [there] would be periods of when I wasn’t really movin’, and it motivated me to get up and make sure I did it more.”*  *“The part that was difficult is we were supposed to be able to share some things. I forget what it’s called on that particular part of the app, that you could remain in contact with the group, you could share recipes or whatever. That part was not working. I couldn’t get it to scroll down so I could read part of it. It was really hard to add things to it.”* |
| MORPH Intervention  Components | Food tracking contributed to adherence and accountability  Mindfulness activities improved awareness of anxiety provoking thoughts | Food tracking *“opened my eyes to what needed to change.”*  *“I learned because I've never counted calories. I never counted fat grams. I never knew how to. I just didn't bother to learn 'cause I've never had to. So, the nutritional part with Bev was fantastic for me. It was very time-consuming to look up in the little book every single thing you put in your mouth all day long. That was a complaint that we all had, but we all understood, or at least I did. I understood the reasoning behind it and the education that I derived from having someone hold my feet to the fire and say, ‘Okay, you have to do this so you can learn.’ That was good.”*  *“…when you start beating yourself up, ‘All right, you ate too much yesterday, and you're way over your allotment,’ or ‘You didn't walk enough yesterday,’ or ‘Darn you. You drank water, but you forgot to record it,’ all that kinda stuff. If you just stop that tape in your head—and I think that's what the mindfulness thing did.”* |
| Group Meetings | Group meetings provided motivational support and accountability  Challenges with the virtual format inhibited some participants from fully accessing social support from group leaders and other participants | *“I liked it if anybody had recipes that they wanted to share, because you come up with different ideas ‘cause you don’t wanna keep eatin’ the same stuff all the time. Or ideas on what they did for exercise or what they did to make sure they got up and did it.”*  *“There were times that, when I was going to get onto the Webex meeting, that I could hear them, but I could not see them. I knew that it was not my computer or my setup, because I work from home and have Webex meetings for my company with no issues. Somewhere, there's a glitch with how it's being done.”* |

Three key themes of the MORPH intervention emerged from the qualitative interviews: MORPH technology (smart scales, Fitbit, MORPH Companion App) facilitated program adherence and accountability; MORPH intervention components (food tracking and mindfulness activities) facilitated program adherence and awareness, respectively; and, group meetings provided motivational support and accountability. Illustrative quotes provide context and support for the themes.
